# Taste characteristics and symbolic metabolites of Rougui tea with different grades in China

**DOI:** 10.1016/j.fochx.2025.102405

**Published:** 2025-03-21

**Authors:** Jianghua Ye, Yangxin Luo, Yuhua Wang, Yulin Wang, Tingting Wang, Yankun Liao, Junbin Gu, Xiaoli Jia, Qi Zhang, Haibin Wang

**Affiliations:** a College of Tea and Food Science, Wuyi University, Wuyishan 354300, China; b College of Life Science, Longyan University, Longyan 364012, China; c College of JunCao Science and Ecology, Fujian Agriculture and Forestry University, Fuzhou 350002, China

**Keywords:** Rougui tea, National standard sample of tea, Sensory evaluation, Taste characteristics, Symbolic metabolites, Cyclo-glycyl-*L*-phenylalanine (PubChem CID: 7076549), Calyxanthone (PubChem CID: 23902332), 3-Hydroxy-*L*-phenylalanine(PubChem CID: 6950578), 2-Furoic acid (PubChem CID: 6919), L-Valine (PubChem CID: 6287), L-Phenylalanine (PubChem CID: 6140), S-Methyl-L-cysteine (PubChem CID: 24417), p-Acetanisidide (PubChem CID: 4105139), DL-Leucine (PubChem CID: 857), Artocarpanone (PubChem CID: 15298902)

## Abstract

The national tea standard sample is an important reference for the evaluation of tea grades. Clarifying the taste characteristics of different grades of Rougui tea (RGT) and its characteristic metabolites is of great significance for the standardization and scientificization of national standard samples. In this study, metabolites of different grades of RGT were analyzed by sensory evaluation and metabolomics, and symbolic metabolites were obtained and verified. The results showed that the higher the RGT grade, the stronger the mellowness and fresh and brisk taste, and the weaker the bitterness and astringency. Cyclo-glycyl-*L*-phenylalanine and calyxanthone can be used as the main symbolic metabolites to evaluate the quality of RGT, and their contents in 15 different grades of RGT verified the conclusion. This study lays an important foundation for the production of national tea standards and the measurement of commercial tea quality.

## Highlights

• There are significant differences in metabolites between different RGT grades.
• The higher the RGT grade, the stronger the mellowness, freshness and brisk taste.
• The higher the RGT grade, the weaker the bitterness and astringency.
• There are 15 symbolic metabolisms that distinguish different RGT grades.
• Cyclo-glycyl-*L*-phenylalanine and calyxanthone are the main symbolic metabolites for RGT.

## 1 Introduction

Wuyi Shan, as a major tea producing area in China, has tea trees that are uniquely rooted in rock crevices, a unique growing environment that produces the characteristic “rocky bone and flower aroma” (Wang et al., 2022a). In Wuyi Shan, Rougui (*Camellia sinensis* cv. *Rouogui*) is the main cultivated tea variety, and its Rougui tea (RGT) is favored by consumers because of its rich and high aroma, mellow and sweet taste (Liang et al., 2024). The cultivation environment of the tea plant, the quality of fresh leaves, and the processing technology all have a profound influence on the quality of RGT, and this view has been widely recognized by scholars (Liu, Chen, Sun, & Ni, 2022, Ye et al., 2024, Zhang, Zhou, Zhang, Zhang, Zhang, & Guo, 2024). Because of this, the quality of RGTs circulating in the market varies, which makes it difficult for consumers to evaluate the quality of tea leaves through first-hand tasting and use it as a basis for their purchasing decisions. In order to effectively standardize tea quality evaluation standards and alleviate consumers' troubles during the purchase process (Yue, Wang, Peng, Li, & Yang, 2023).

In 2006, the General Administration of Quality Supervision, Inspection and Quarantine (AQSIQ) and the Standardization Administration of China (SAC) jointly promulgated the national standard of “Product of geographical indication-Wuyi rock-essence tea”. The standard not only stipulates the sensory evaluation method of Wuyi rock tea, but also produces a national standard sample (GB/T 18745–2006), which is regularly replaced. However, the current sensory evaluation method mainly relies on the sensory experience of professional judges, which can reflect the quality of tea to a certain extent, but it is difficult to avoid subjectivity and individual differences, and cannot achieve objective evaluation. Therefore, it is of great significance to analyze the national standard samples of different grades in depth from the material level, and to excavate and identify the characteristic signature metabolites for the construction of more refined, objective and reliable national standard samples of RGT.

RGT, as a kind of oolong tea, and for oolong tea, flavor is one of the important quality evaluation indexes, which plays a key role in tea classification (Cai et al., 2022). According to the current national standard of the People's Republic of China “Methodology for sensory evaluation of tea” (GB/T 23776–2018), among the evaluation factors of oolong tea, the proportion of flavor is the largest, reaching 35 %, highlighting its importance. Mmetabolomics technology provides a powerful tool for in-depth analysis of the metabolite content in tea, and then evaluates the flavor characteristics of tea. Through this technique, key compounds that determine the flavor characteristics of tea can be obtained. For example, Huang et al. (2021) used metabolomics to analyze the effect of storage age on the taste characteristics of black tea, and found that bitterness and freshness of black tea were negatively correlated with storage year, and identified seven symbolic metabolites that could be used to determine black tea vintage. Similarly, Zeng et al. (2023) combined sensory evaluation and metabolomics approaches to analyze different grades of Tieguanyin tea, and found five symbolic metabolites determining differences in Tieguanyin grades, and the content of these metabolites became an effective indicator for evaluating tea grades. In addition, Zhou et al. (2024) analyzed the metabolites of Tangyang Congou black tea of different grades, and found that the higher tea grade, the stronger the taste characteristics, and the key symbolic metabolites that determine these taste characteristics are mainly four amino acids. It can be seen that the metabolite analysis of different grades of tea can clarify the reasons for the differences in the taste characteristics of different grades of tea, and identify the symbolic metabolites for determining the grade of tea.

The grading of RGT, as the famous Wuyi rock tea, still relies on the comparison of sensory evaluation with the national RGT standard sample, a method that lacks objectivity and validity. The present study was to explore the reasons for the formation of flavor characteristics of different grades of RGT and to obtain symbolic metabolites that can objectively evaluate different grades of RGT, with a view to laying a foundation for the scientific development of Chinese RGT standard samples. Accordingly, the present study was conducted to analyze the taste characteristics of different grades of RGT by combining sensory evaluation with ultra-high performance liquid chromatography-mass spectrometry in series (UPLC-MS/MS) using standard samples of Chinese national RGT (super-grade RGT, first-grade RGT, and second-grade RGT) as materials. The flavor characteristics of different grades of RGT were analyzed to clearly distinguish the main symbolic metabolites of different grades of RGT and their contributions to flavor characteristics, and to obtain symbolic metabolites that can objectively and effectively determine the grade of RGT. On the basis of this study, 15 RGTs of different grades were selected through sensory evaluation, with 5 samples for each grade, and the content of the symbolic metabolites was determined to verify the aforementioned conclusions. This study lays an important foundation for the establishment of a national standard sample of RGT and the construction of a tea grade evaluation system in China.

## 2 Materials and methods

### 2.1 Preparation of RGT national standard samples

Three grades of RGT national standard samples were selected in this study, namely super-grade RGT (R3), first-grade RGT (R2) and second-grade RGT (R1). The samples were produced by the Food and Drug Administration of Wuyishan City, Fujian Province, China and the Wuyishan Tea Association. The RGT national standard sample is made in strict accordance with the national standards, “Product of geographical indication-Wuyi rock-essence tea” (GB/T 18745–2006) and “Directives for the work of reference materials” (GB/T 15000), and the grade evaluation is in strict accordance with the national standard, “Methodology for sensory evaluation of tea” (GB/T 23745–2006) oolong tea evaluation method. The validity period of RGT national standard samples is from December 3, 2022 to December 2, 2025. The obtained RGT standard samples of different grades are stored at −20 °C for subsequent experiments.

### 2.2 Chemicals and instrumentation for experiments

The methanol, formic acid, acetonitrile and polypropylene glycol used in this study were chromatographically pure grade and purchased from China National Pharmaceutical Group (Beijing, China). The standards of cycloglycyl-*L*-phenylalanine, calyxanthone and other compounds were purchased from Sigma-Aldrich (Shanghai, China). The main instrumentation included vacuum freeze dryer model Scientz-100F (Zhejiang, China), UPLC model Nexera X2 (Shimadzu, Kyoto, Japan), and MS/MS model 4500 QTRAP (Applied Biosystems, MA, USA).

### 2.3 Sensory evaluation method for RGT national standard samples

The method for sensory evaluation of different grades of RGT is strictly in accordance with the national standard, “Methodology for sensory evaluation of tea” (GB/T 23776–2018) Oolong tea evaluation method. Briefly, nine professional reviewers with the qualification of China's first-class tea appraiser conducted sensory evaluation for different grades of RGT, mainly evaluating five indexes such as tea shape, liquor color, aroma, taste and foliage fundus. Each index was scored according to a percentage system, and then converted according to the coefficients of each index in GB/T 23776–2018 in the sensory review of oolong tea to obtain the total evaluation score. The coefficients of tea shape, liquor color, aroma, taste, and foliage fundus are 20 %, 5 %, 30 %, 35 % and 10 %, respectively.

### 2.4 Extraction of metabolites from the national standard sample of RGT

The RGT samples of each grade were dried separately using a vacuum freeze dryer and ground to powder form. Subsequently, 100 mg of tea samples were weighed into a 10 mL centrifuge tube, to which 2.5 mL of 70 % methanol solution pre-cooled to −20 °C was added. A vortex oscillator was used to oscillate and mix the mixture, which was performed by vortex oscillation every 30 min for 30 s for a total of 6 oscillations and then centrifuged to collect the resulting supernatant. After that, 1 mL of 70 % methanol solution pre-cooled to −20 °C was added to the precipitate again, and the above steps of oscillation and centrifugation were repeated to collect the supernatant, and then the extraction was repeated once more. The supernatants obtained from the three extractions were combined and filtered through a 0.22 μm microporous filter membrane, and the final filtrate obtained was used for UPLC-MS/MS analytical tests. For different grades of RGT samples, each sample was subjected to three independent replicates and each independent replicate was averaged from three parallel tests.

### 2.5 Determination and analysis of RGT national standard sample by using UPLC-MS/MS

The determination of tea metabolites was referred to the method of Ye et al. (2024). For UPLC analysis, an Agilent SB-C18 column (1.8 μm, 2.1 mm × 100 mm) was used. The mobile phase was divided into phase A and phase B. Phase A was ultrapure water containing 0.1 % formic acid, and phase B was acetonitrile. The elution gradient for UPLC was set at 19:1 for A:B at 0 min, linearly decreased to 1:19 from 0 to 9 min, maintained at 1:19 from 9 to 10 min, linearly increased to 19:1 from 10 to 11 min, and maintained at 19:1 after 11 min. The injection volume was 2 μL, the flow rate was 35 mL/min, and the column temperature was 40 °C. For MS/MS analysis, the electrospray ionization temperature was set at 550 °C, and the spray voltages for positive and negative ions were adjusted to 5500 and − 4500 V, respectively. The ion source I and II pressures were set to 50 psi and 60 psi, respectively, and the air curtain gas pressure was 25 psi. The multiple reaction monitoring (MRM) mode was used, and for each metabolite-specific ion pair, the corresponding declustering potential and collision energy were set. Before scanning, the instrument was tuned and mass calibrated using polypropylene glycol solutions (10 and 100 μmol/L). After GC–MS analysis, the raw data of the samples were collected using Analyst software (version 1.6.3) and processed by MassHunter software (B 08.00) for subsequent qualitative and quantitative analysis (Wen et al., 2024). Two to three qualitative ions were selected for each compound and compared with the NIST20 mass spectrometry database, and if the retention time of the detection was consistent with the standard reference and all selected ions appeared in the mass spectra of the samples after deduction of the background, it was determined to be the substance. 3-Hexanone-2,2,4,4-d4 (CAS 24588–54-3) was used as an isotopic internal standard. One quantitative ion was selected for each compound for integral correction, and the quantitative analysis of the compounds was realized by converting the peak area to compare with the internal standard.

### 2.6 Sensory evaluation of 15 different grades of RGT and determination of symbolic metabolites

Based on the above findings, cycloglycyl-*L*-phenylalanine and calyxanthone might be the symbolic metabolites that distinguished different grades of RGT. Accordingly, five samples for each of different grades of RGTs (super-grade RGT, first-grade RGT, and second-grade RGT), totaling 15, were collected in this study. The sensory evaluation was carried out in strict accordance with the national standard of “Methodology for sensory evaluation of tea” (GB/T 23776–2018) Oolong tea evaluation method, as described in 2.3. At the same time, metabolites of 15 different grades of RGT were extracted and the contents of cycloglycyl-*L*-phenylalanine and calyxanthone were determined. Specific extraction and determination methods were described in 2.4 and 2.5.

### 2.7 Statistical analysis

Excel 2020 was used to perform preliminary statistics on the raw data of RGT sensory evaluation and the metabolome raw data from this study. All data were further analyzed and graphically produced using Rstudio software (v 4.2.3) (Wickham, 2019). Differences between samples were analyzed using paired Student ‘s *t*-tests, with *p* < 0.05 being taken as a significant level of difference. For generating radar plots of the RGT sensory evaluation data, the R package used was fmsb 0.7.6. To visualize the distribution of RGT metabolites, gghalves 0.1.4 were used to draw box plots. The networkD3 package was selected to create mulberry plots of RGT metabolites for analysis of their classification and content. For volcano plots of different classes of RGT, ggplot2 3.5.0 was used. The two powerful R packages, ropls and mixOmics, were combined in the construction of the OPLS-DA (Orthogonal Partial Least Squares Discriminant Analysis) model to differentiate between different grades of RGT. To present bubble feature plots of RGT metabolites, ggplot2 3.4.0 was selected. Stats 4.2.0 was used to perform K-means clustering analysis of RGT differential metabolites. For TOPSIS analysis to assess the contribution of RGT metabolites, dplyr 1.1.4 was used. Microeco was then used to perform the Kruskal-Wallis test analysis of the different metabolites in the RGT and to generate the corresponding graphs. Finally, in order to visualize the content of RGT metabolites in relation to taste, the pheatmap 1.0.12 package was used to produce heat maps.

## 3 Results and discussion

### 3.1 Sensory evaluation of different grades of RGT

**Fig. 1:**
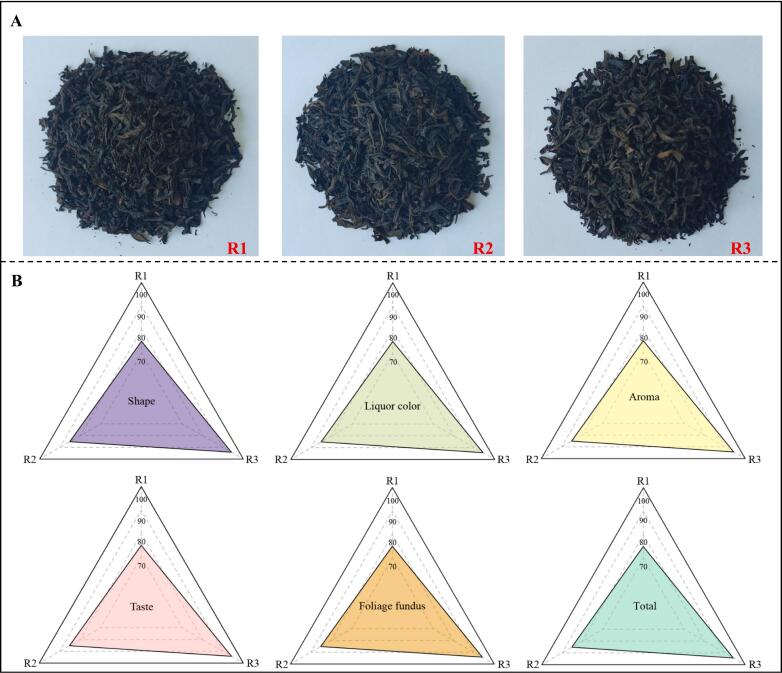
National standard samples of different grades of RGT and analysis of sensory evaluation. Note: R1: Second-grade RGT, R2: First-grade RGT, R3: Super-grade RGT; A: National standard samples of different grades of RGT; B: Sensory evaluation of different grades of RGT.

Sensory evaluation is an important method for determining tea quality (Lin et al., 2024). In this study, different grades of RGT (Fig. 1A) were assessed in sensory evaluation. The results showed (Fig. 1B) that the shape, liquor color, aroma, taste and foliage fundus of R1 were 75.04, 75.09, 75.07, 75.12 and 75.01 points, respectively, with a total overall evaluation score of 75.07; those of R2 were 85.22, 85.12, 85.13, 85.19 and 85.19 points, respectively, with a total overall rating of 85.17; and 94.14, 94.28, 94.33, 94.57 and 94.19 points for R3, with a total overall rating of 94.30. The evaluation results of each index showed that R3 had the best quality, R2 was second and R1 was the worst. It can be seen that the shape of the high-quality RGT was tight and even, the foliage fundus was fat and soft, the liquor color was golden and bright, the aroma was rich and the taste was mellow.

### 3.2 Metabolite analysis of different grades of RGT

**Fig. 2:**
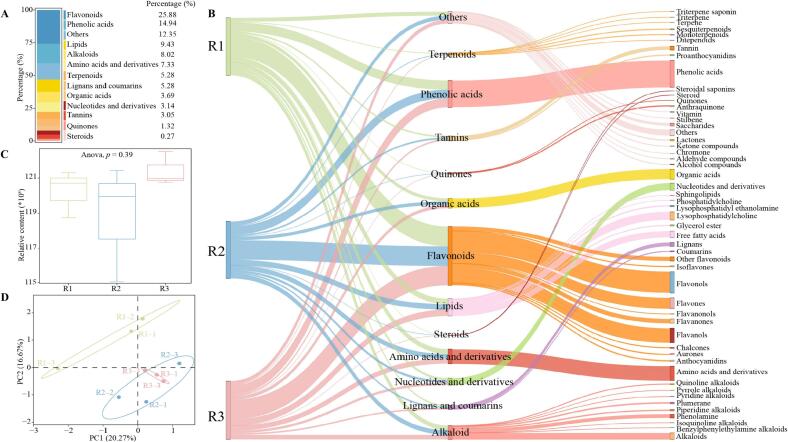
Metabolite analysis of different grades of RGT. Note: R1: Second-grade RGT, R2: First-grade RGT, R3: Super-grade RGT; A: Quantitative analysis of different classes of metabolites, B: Classification and content analysis of metabolites of different groups of RGT, C: Total metabolite amount analysis of different groups of RGT, and D: Principal component analysis with metabolite content of different groups of RGT.

In the sensory evaluation of tea, taste is one of the most important indexes for evaluating tea quality (Wang et al., 2022b). Taste also accounts for the largest proportion of the indexes in the sensory evaluation of RGT (Zhang, Cao, Granato, Xu, & Ho, 2020). And, the intensity of different taste characteristics of tea is closely related to the metabolite content in its leaves, especially the content of key metabolites (Feng et al., 2024). In this study, broadly targeted metabolomics technology was used to determine the metabolites of different grades of RGT, and the results (Table S1, Table S2, Table S3, Fig. 2A) showed that a total of 2195 metabolites were detected, which could be categorized into 13 groups according to the first level of classification, which were flavonoids (25.88 %), phenolic acids (14.94 %), lipids (9.43 %), alkaloids (8.02 %), amino acids and derivatives (7.33 %), terpenoids (5.28 %), lignans and coumarins (5.28 %), organic acids (3.69 %), nucleotides and derivatives (3.14 %), tannins (3.05 %), quinones (1.32 %), steroids (0.27 %) and others (12.35 %)。 It is evident that among the metabolites of different grades of RGT, flavonoids accounted for the highest percentage of 25.88 %, followed by phenolic acids. Further analysis revealed (Fig. 2B) that 2195 metabolites, when categorized according to primary and secondary classification, still showed the highest content of flavonoids, followed by phenolic acids, in terms of content in the primary classification, and the highest content of phenolic acids, followed by flavonols, in the secondary classification. In addition, analysis of total metabolite content revealed (Fig. 2C) that there was no significant difference in total metabolite content between the different grades of RGT (*p* = 0.39). Principal component analysis of different metabolite contents revealed (Fig. 2D) that R2 and R3 were more similar, while there was an obvious difference with R1. It is evident that the differences in total metabolite content between different grades of RGT were not significant, but there might be significant differences in the content of different metabolites, which in turn affected tea quality.

### 3.3 Screening of key differential metabolites in different grades of RGT

**Fig. 3:**
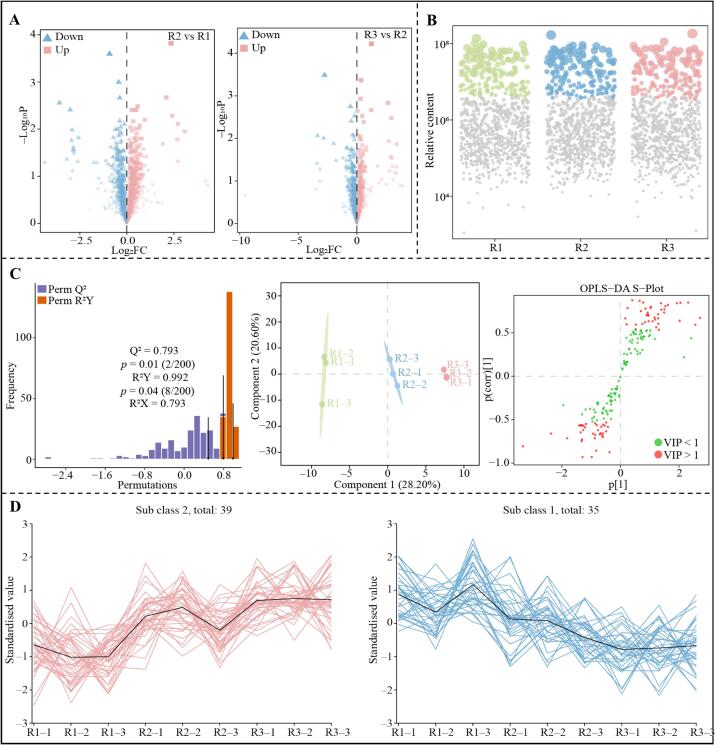
Screening of key differential metabolites of different grades of RGT.

The quality of different teas is related to the content of various metabolites they contain, especially some key metabolites that differ significantly between different teas and whose content contributes significantly to the formation of tea taste characteristics (Yue et al., 2023). For example, Shan et al. (2024) found that there were 31 key differential metabolites, mainly flavonol glycosides, amino acids, and phenolic acids, that affected the taste characteristics of green tea. The sweetness, mellowness, and freshness of high-quality black tea are stronger, and the key reason is that its content of 13 key metabolites, which are mainly amino acids and theaflavins, is higher than that of low-quality black tea (Yue et al., 2024). Accordingly, the metabolite content of different grades of RGT was determined in this study, and then volcano plots were used to screen metabolites with significant differences between different grades of RGT, and the results showed (Fig. 3A) that 803 metabolites out of 2195 metabolites were significantly different, of which 396 metabolites showed an increasing trend in their content with increasing RGT grade, and 407 metabolites showed a decreasing trend. Further analysis of 803 significantly different metabolites using bubble characterization plots revealed (Fig. 3B) that a total of 171 characteristic metabolites were obtained, 88 of which showed an increasing trend in content and 83 showed a decreasing trend with increasing RGT grade.

**Fig. 4:**
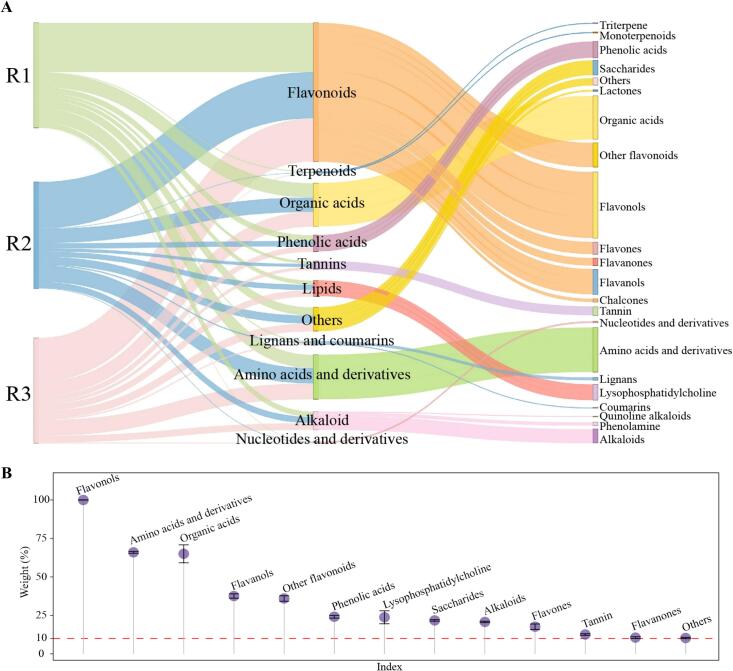
Classification, content and its contribution analysis of key differential metabolites in different grades of RGT. Note: R1: Second-grade RGT, R2: First-grade RGT, R3: Super-grade RGT; A: Classification of key differential metabolites of different grades of RGT and their content analysis, B: TOPSIS analysis of the contribution of key differential metabolites in distinguishing different groups of RGT after classification.

Note: R1: Second-grade RGT, R2: First-grade RGT, R3: Super-grade RGT; A: Volcano gram analysis of metabolites from different grades of RGT to screen for significantly different metabolites; B: Bubble feature plot of metabolites with significant differences in different grades of RGT to screen for characteristic metabolites; C: OPLS-DA model construction of characteristic metabolites of R1, R2, and R3 to screen for key metabolites; D: K-means clustering analysis of key metabolites.

On the basis of the above studies, the present study further explored and constructed an OPLS-DA model for R1, R2 and R3 by using the obtained characteristic metabolites, aiming to screen the key metabolites to distinguish the different classes of RGTs. The results showed (Fig. 3C) that the constructed model was able to accurately classify R1, R2 and R3 into different regions. The fit (R^2^Y = 0.992, *p* = 0.04) and predictability (Q^2^ = 0.793, *p* = 0.01) of the model were extremely high and both reached the significant level. The in-depth analysis of the model yielded the importance eigenvalues (VIP) for 171 characteristic metabolites, of which 74 metabolites were determined to be key metabolites capable of significantly differentiating between R1, R2, and R3 due to their VIP values greater than one. K-means clustering analysis of the above key metabolites revealed (Fig. 3D) that the content of 39 metabolites showed an increasing trend and the content of 35 metabolites showed a decreasing trend with the increase of RGT grade. Classification and content analysis of these 74 key metabolites revealed (Fig. 4A) that according to the primary classification, they could be categorized into 11 groups, with the top three most abundant metabolites being flavonoids, amino acids and derivatives and organic acids; according to the secondary classification, they could be categorized into 22 groups, with the top three most abundant metabolites being flavonoids, amino acids and derivatives and organic acids. Based on the secondary classification of 74 key metabolites, TOPSIS was used to analyze the contribution weights of different classes of metabolites in distinguishing R1, R2 and R3, and the results revealed (Fig. 4B) that 13 classes of metabolites contributed more than 10 %, namely, flavonols (99.58 %), amino acids and derivatives (65.98 %), organic acids (65.03 %), flavanols (37.56 %), other flavonoids (35.99 %), phenolic acids (24.12 %), lysophosphatidylcholine (23.79 %), saccharides (21.73 %), alkaloids (20.75 %), flavones (17.66 %), tannin (12.59 %), flavanones (10.58 %) and others (10.30 %). It is evident that the metabolites that played an important role in distinguishing between different classes of RGT were mainly flavonols, amino acids and derivatives and organic acids.

### 3.4 Symbolic metabolites of different classes of RGT and their taste characteristics

**Fig. 5:**
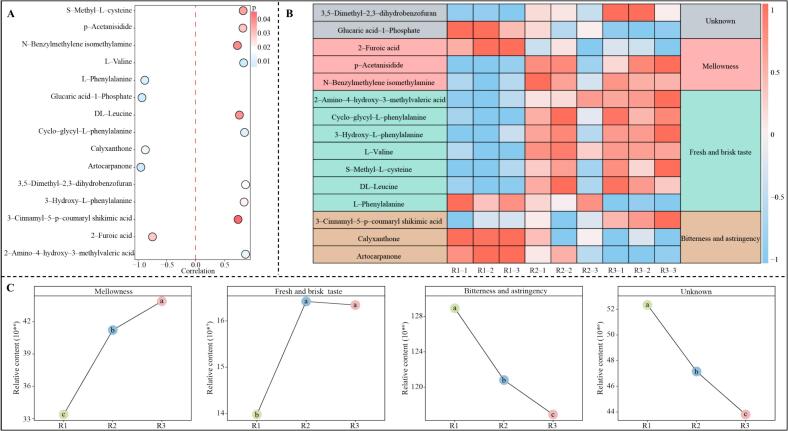
Screening of symbolic metabolites in different grades of RGT and their taste characteristics. Note: R1: Second-grade RGT, R2: First-grade RGT, R3: Super-grade RGT; A: Kruskal-Wallis test to screen symbolic metabolites of different grades of RGT, B: Taste characteristics of symbolic metabolites, C: The intensity of taste characteristics of symbolic metabolites in different grades of RGT. Different lower case letters indicate that the differences between different samples reached the *p* < 0.05 level.

Based on the previous analysis, 74 key metabolites distinguishing R1, R2 and R3 were obtained in the present study, of which 13 groups of metabolites with a contribution of more than 10 % were identified, especially flavonols, amino acids and derivatives and organic acids. It has been reported that high contributing metabolites in different quality teas may contain symbolic metabolites that distinguish different grades of tea (Tian et al., 2024). These symbolic metabolites are important for the formation of special flavors and characterization of tea quality (Zhu et al., 2024). Accordingly, the Kruskal-Wallis test was used in this study to analyze the key metabolites distinguishing different grades of RGT, and a total of 15 symbolic metabolites were obtained (Fig. 5A). Among them, the contents of 10 symbolic metabolites, which showed an increasing trend with increasing RGT grade, were 2-amino-4-hydroxy-3-methylvaleric acid, 3,5-dimethyl-2,3-dihydrobenzofuran, cycloglycyl-*L*-phenylalanine, 3-hydroxy-*L*-phenylalanine, L-valine, *S*-methyl-L-cysteine, p-acetanisidide, DL-leucine, 3-cinnamyl-5-p-coumaryl shikimic acid and *N*-benzylmethylene isomethylamine. The contents of the five symbolic metabolites, on the other hand, showed a decreasing trend, namely 2-furoic acid, calyxanthone, *L*-phenylalanine, glucaric acid-1-phosphate and artocarpanone. Further analysis of the taste characteristics presented by the 15 symbolic metabolites revealed (Fig. 5B) that these symbolic metabolites presented three main taste characteristics, namely mellowness, fresh and brisk taste and bitterness and astringency. The analysis of taste characteristics intensity showed (Fig. 5C) that the intensity of mellowness and fresh and brisk taste tended to increase significantly with increasing RGT grade, while the intensity of bitterness and astringency tended to decrease significantly. Among the tea taste characteristics, mellowness and fresh and brisk taste are preferred by consumers, while bitterness and astringency are not easily accepted by consumers (Deng et al., 2022). Therefore, reducing bitterness and astringency and enhancing mellowness and fresh and brisk taste of tea during tea processing can improve tea quality (Cao et al., 2021; Sun et al., 2023). This study found that the differences in taste characteristics among different grades of RGT were mainly bitterness and astringency, mellowness, and fresh and brisk taste. The higher the quality grade of RGT, the stronger the mellowness and fresh and brisk taste, and the weaker the bitterness and astringency, and the main determinant of the intensity of these taste characteristics was the content of the 15 symbolic metabolites.

### 3.5 Contribution of symbolic metabolites in differentiating different grades of RGT and their validation analysis

**Fig. 6:**
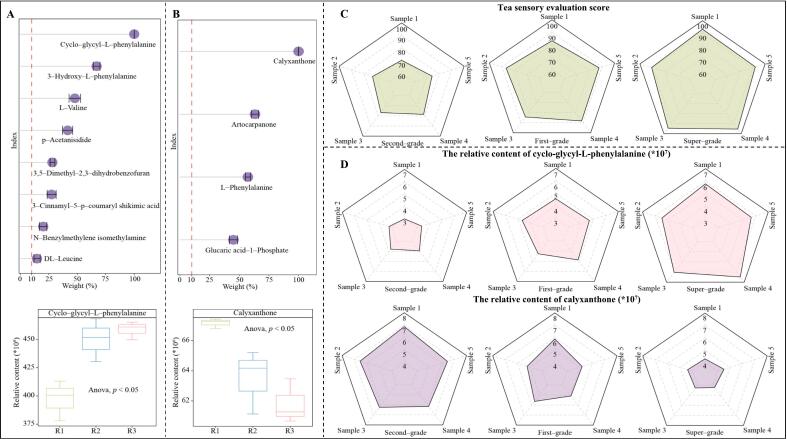
Contribution of symbolic metabolites in distinguishing different grades of RGT and their validation analysis. Note: R1: Second-grade RGT, R2: First-grade RGT, R3: Super-grade RGT; A: TOPSIS analysis of 10 symbolic metabolites significantly and positively correlated with RGT grades; B: TOPSIS analysis of 5 symbolic metabolites significantly and negatively correlated with RGT grades; C: Sensory evaluation scores of 15 different grades of RGT; D: The content of cycloglycyl-*L*-phenylalanine and calyxanthone in 15 different grades of RGT.

Symbolic metabolites play an important role in the evaluation of tea grades. Zeng et al. (2023) used a combination of sensory evaluation and metabolomics to analyze different grades of Tieguanyin, and found that five symbolic metabolites existed in different grades of Tieguanyin, and tea grades could be determined by their content. Gao et al. (2022) analyzed different grades of Panyong black tea and found that there were six main symbolic metabolites that distinguished different grades of tea. Based on the previous analysis, the present study further analyzed the contribution of symbolic metabolites in distinguishing different RGT grades by using TOPSIS, and the results revealed (Fig. 6A) that among the 10 symbolic metabolites that showed a significant increase with increasing RGT grade, the top three contributors were cycloglycyl-*L*-phenylalanine (99.58 %), 3-hydroxy-*L*-phenylalanine (66.99 %) and L-valine (47.88 %), while among the 5 symbolic metabolites that showed a significant decrease with increasing RGT grade (Fig. 6B), the top three contributors were calyxanthone (99.32 %), artocarpanone (63.27 %) and *L*-phenylalanine (57.29 %). It is evident that cycloglycyl-*L*-phenylalanine and calyxanthone may play an important role in differentiating between different grades of RGT. Accordingly, a total of fifteen RGTs, their sensory evaluation of the second-grade RGT (5 samples), first-grade RGT (5 samples), and super-grade RGT (5 samples), were again selected for this study, and the distribution of RGT scores for each grade ranged from 70.14 to 78.52, 83.27 to 88.28, and 83.27 to 88.28, with mean scores of 74.62, 86.01, and 93.21, respectively (Fig. 6C). The cycloglycyl-*L*-phenylalanine and calyxanthone contents of 15 RGTs with different grades were further determined, and the results found (Fig. 6D) that the cycloglycyl-*L*-phenylalanine content tended to increase significantly with the increase of the RGT grade, while the calyxanthone content showed a significant decreasing trend. It is evident that these two symbolic metabolites play an important role in distinguishing different grades of RGT and can be used to evaluate the quality of RGT.

## 4 Conclusion

**Fig. 7:**
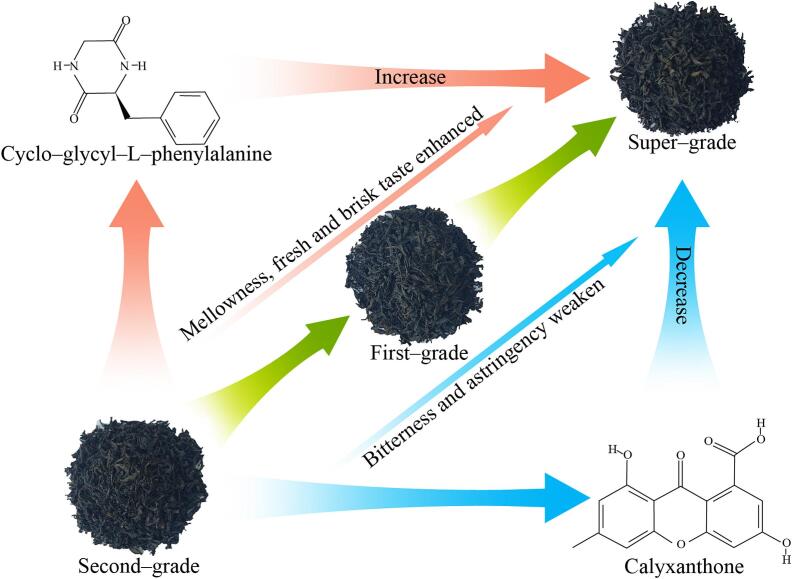
Representative metabolites affecting RGT quality.

In this study, the key reasons for the formation of quality differences between different grades of RGT were analyzed using sensory evaluation combined with metabolomics, and symbolic metabolites affecting quality differences were screened and validated. Seventy-four key metabolites were found to distinguish between different grades of RGT, and the metabolites that played a major role were flavonols, amino acids and derivatives, and organic acids. Based on the above key metabolites, further analysis revealed that there were 15 symbolic metabolites distinguishing different grades of RGT, presenting taste characteristics mainly mellowness, fresh and brisk taste and bitterness and astringency. The higher the quality level of RGT, the stronger the mellowness and fresh and brisk taste, and the weaker the bitterness and astringency. Cyclo-glycyl-*L*-phenylalanine and calyxanthone could be used as the main symbolic metabolites to evaluate the quality of Rougui (Fig. 7). The validation results of 15 samples of different grades of RGT again showed that the higher the quality of RGT, the more cycloglycyl-*L*-phenylalanine and the less calyxanthone it contained. In this study, by analyzing the differences in metabolites of different grades of RGT, symbolic metabolites characterizing the quality of RGT were screened and obtained and validated. This study lays an important foundation for the refinement of RGT national tea standard samples and the grading of RGT commercial tea.

## CRediT authorship contribution statement

**Jianghua Ye:** Writing – original draft, Visualization, Methodology, Funding acquisition, Data curation, Conceptualization. **Yangxin Luo:** Writing – original draft, Visualization, Methodology, Funding acquisition, Data curation, Conceptualization. **Yuhua Wang:** Writing – original draft, Visualization, Methodology, Funding acquisition, Data curation, Conceptualization. **Yulin Wang:** Writing – original draft, Visualization, Methodology, Formal analysis. **Tingting Wang:** Writing – original draft, Visualization, Methodology, Formal analysis. **Yankun Liao:** Writing – original draft, Visualization, Methodology, Formal analysis. **Junbin Gu:** Writing – original draft, Visualization, Methodology, Formal analysis. **Xiaoli Jia:** Writing – review & editing, Validation, Formal analysis. **Qi Zhang:** Writing – review & editing, Validation, Formal analysis. **Haibin Wang:** Writing – review & editing, Writing – original draft, Supervision, Resources, Project administration, Methodology, Funding acquisition, Conceptualization.

## Institutional review board statement

The study was conducted in accordance with the Declaration of Helsinki, and approved by the Institutional Review Board of Wuyi University (No. 20231218028 and 18 December 2023).

## Declaration of competing interest

The authors declare that they have no known competing financial interests or personal relationships that could have appeared to influence the work reported in this paper.

## Data Availability

Data will be made available on request.

## Appendix A Supplementary data

Supplementary material 1 Table S1. Metabolite raw data for second-grade RGT. Table S2. Metabolite raw data for first-grade RGT. Table S3. Metabolite raw data for super-grade RGT. Image 1
